# Provider Perspectives on the Use of Mental Health Apps, and the BritePath App in Particular, With Adolescents at Risk for Suicidal Behavior: Qualitative Study

**DOI:** 10.2196/64867

**Published:** 2025-02-26

**Authors:** Frances Lynch, Julie Cavese, Lucy Fulton, Nancy Vuckovic, David Brent

**Affiliations:** 1 Kaiser Permanente Center for Health Research Portland, OR United States; 2 Cavese Counseling Portland, OR United States; 3 Vuckovic Consulting Vuckovic Consulting Portland, OR United States; 4 Department of Psychiatry University of Pittsburgh Medical Center Pittsburgh, PA United States

**Keywords:** depression, adolescent, suicidality, safety plan, mental health, apps, suicide

## Abstract

**Background:**

Many youth with significant mental health concerns face limited access to mental health services. Digital programs, such as mobile apps designed to address mental health issues, have the potential to expand access to strategies for managing these conditions. However, few mental health apps are specifically designed for youth experiencing severe concerns, such as suicidal ideation. BritePath is a new app developed to enhance communication and interaction between providers and youth at risk for suicidal behavior.

**Objective:**

This study aims to explore health care providers’ opinions and concerns regarding the use of mental health apps for youth at significant risk of suicidal behavior.

**Methods:**

We conducted individual semistructured interviews with 17 providers across 7 states. Interviews were conducted via video, recorded, and transcribed. Codes were developed using a team-based approach, with discrepancies resolved through team discussions.

**Results:**

Most providers were aware of mental health apps in general and expressed interest in trying the BritePath app with patients experiencing depression, suicidality, or both. Analyses identified 4 key themes related to mental health apps: (1) almost all providers viewed mental health apps as an adjunct to, rather than a replacement for, psychotherapy visits; (2) most providers were concerned about the cost of apps and youth access to them; (3) providers noted the challenge of maintaining patient engagement with apps over time; and (4) providers were concerned about patient privacy, in terms of both data shared with app developers and data privacy within families. Analyses of providers’ opinions specifically about the BritePath app identified 4 additional themes: (1) providers believed that access to safety plans within BritePath could be beneficial for youth at risk for suicidal behavior; (2) providers reported that BritePath’s interactive features could enhance communication between providers and youth; (3) providers appreciated BritePath’s flexibility and the ability for both youth and providers to tailor its content to individual needs; and (4) providers expressed concerns about integrating BritePath into clinical workflows within health systems.

**Conclusions:**

The use of mental health apps is expanding, yet there is limited understanding of how to effectively integrate these tools into mental health treatment. Providers are increasingly referring patients to mental health apps, and most expressed interest in trying the BritePath app for patients with depression, suicidality, or both. However, providers also identified several concerns, particularly regarding privacy and safety.

## Introduction

### Background

Adolescent suicidal behavior, suicide ideation, and depression are major public health problems that have increased significantly over the past 20 years [1-3] and have been exacerbated by the COVID-19 pandemic and its sequelae [4-8]. For example, between 2019 and 2021, the number of female high school students reporting that they had seriously considered attempting suicide increased by 6% [3]. These increases in suicidal ideation, behavior, and depression have disproportionately affected youth from historically minoritized racial and ethnic groups [2-4]. Once youth are identified as having a significant risk of suicidal behavior, they are typically treated in emergency departments or urgent care settings or hospitalized for stabilization before beginning outpatient treatment [9]. However, many youth remain at high risk of suicidal behavior even after acute treatment or stabilization in intensive settings. Managing youth with high levels of suicidal risk requires close communication between patients and clinical providers, such as psychiatrists or mental health therapists (referred to collectively as providers hereafter). Evidence-based care for these youth includes regular monitoring of depression symptoms, suicidal ideation, and suicidal behavior [9-11]. However, most health systems fall short of maintaining this level of close communication due to barriers such as lack of time and resources, as well as difficulties in staying in touch with at-risk youth after they leave intensive settings or acute treatment.

To address these concerns, researchers are developing new mobile apps aimed at improving both the efficiency of communication with youth at risk for suicidal behavior and shared decision-making between providers and adolescent patients. These tools are increasingly promoted to address mental health concerns in general and depression in particular [12-16]. Empirically based apps for depression range from cognitive behavioral self-help programs [15,16] to screening [16,17] or mood-tracking apps [18]. Recent research indicates that these tools are generally acceptable to providers [13,17]. However, there are several limitations to their use in clinical practice. Much of the evidence comes from surveys asking providers about the acceptability of apps [19] or studies that have not focused on specific concerns about particular apps [13]. Few studies have examined the use of apps within the context of mental health treatment [15]; instead, most have focused on patients’ use of apps independent of providers [13]. To date, no prior research has examined the acceptability of apps designed to support shared decision-making between providers and youth at high risk for suicidal behavior. While some experts have raised potential ethical and safety concerns about using digital interventions with high-risk populations [19,20], improving connections to treatment after nonfatal suicidal behavior has the potential to enhance the treatment trajectory and long-term outcomes for these youth.

### Study Goal

The aims of this study were to characterize providers’ (1) opinions on the barriers and benefits of using apps in mental health care in general, (2) perspectives on the use of apps with adolescents with depression or suicidal thoughts and young adult patients, (3) interest in and willingness to use a recently developed app (BritePath) intended for use with adolescents with suicidal thoughts or young adult patients, and (4) barriers to implementing the BritePath app in routine clinical practice.

## Methods

### Implementation of BritePath: Benefits and Barriers

This qualitative study was conducted as part of the Center for Enhancing Treatment and Utilization for Depression and Emergent Suicidality (ETUDES Center), an ALACRITY Center funded by the National Institute of Mental Health (NIMH P50MH115838), aimed at helping providers better support youth experiencing severe depression, suicidality, or both. This study explores issues related to the use of mental health apps in general with this population and examines potential benefits and barriers to implementing an already developed app, BritePath, in health systems that have not participated in the research studies developing BritePath and are not affiliated with the ETUDES Center. To date, all information regarding the acceptability of the BritePath app has been gathered within the institutions that developed it.

### The BritePath App

The BritePath app was developed to improve communication between providers and youth with depression or suicidal thoughts. It consists of 3 integrated components: (1) *Guide2BRITE*, an electronic guide for mental health providers that includes step-by-step instructions for onboarding a patient’s safety plan into the app, as well as guidance on discussing key treatment components such as emotion regulation and distress tolerance skills; (2) the *BRITE app*, a personalized and interactive safety plan and self-monitoring tool for youth; and (3) the clinician dashboard, *BRITEBoard*, which allows providers to track youth’s app use, distress levels, and treatment progress while facilitating communication and collaboration among mental health and primary care providers.

**Figure 1:**
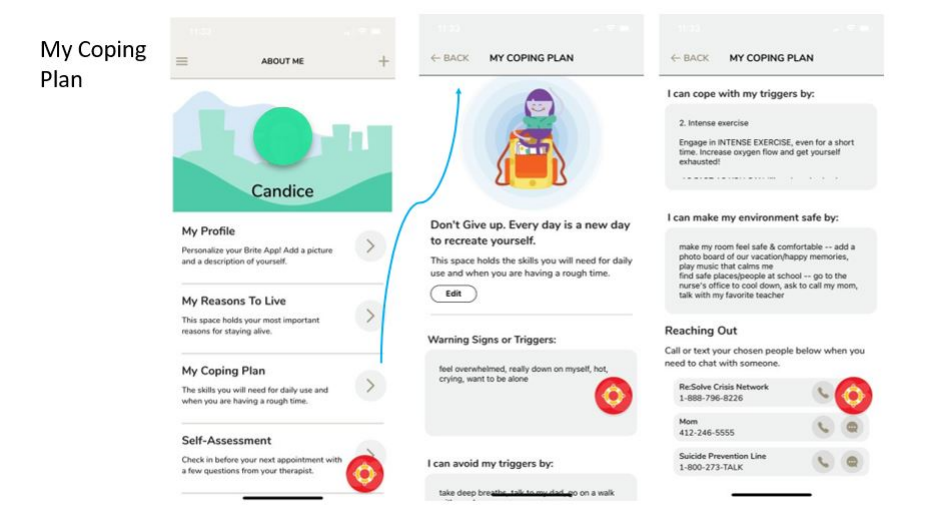
Example of the safety plan in the BritePath app.

BritePath promotes self-monitoring and self-management through personalized strategies to avoid or cope with triggers for suicidal urges [21]. It is based on BRITE, a patient-facing safety planning app developed by researchers in psychiatry and psychology at the University of Pittsburgh and the University of Texas Southwestern Medical Center [21]. To our knowledge, BritePath is the first smartphone-based safety planning app to be tested in clinical trials involving adolescents with severe depression or suicidal thoughts. BritePath provides providers with tools to onboard their adolescent and young adult patients to the app. This includes assisting youth in developing and personalizing a safety plan, incorporating distress tolerance and emotion regulation strategies, and monitoring symptomatic progress for at-risk youth in their care. Figure 1 provides a screenshot from the BritePath app for a mock patient.

BritePath was first tested for clinical effectiveness in a pilot randomized trial that evaluated BritePath in combination with a related intervention. The study found that the combined intervention reduced the rate of suicide attempts by 50% in the 6 months following hospital discharge [21]. This pilot study also suggested that more frequent use of BritePath increased reasons for living. However, the small sample size did not allow for comprehensive testing of BritePath alone. A more recent, larger randomized trial found that BritePath is as effective as usual care for youth hospitalized for suicidality. Additionally, youth who received BritePath were less likely to have a subsequent suicide attempt and had a longer time until a repeat attempt [22].

### Sampling and Recruitment

We identified a convenience sample of mental health and primary care providers who have cared for patients with mental health concerns. Providers were recruited through health systems participating in the Mental Health Research Network [23] and via snowball sampling [24,25], in which interviewed providers recommended additional potential participants. No specific level of digital experience or skills was required for participation in the study.

### Ethical Approval

All study procedures were approved by the Institutional Review Board of the Kaiser Permanente Center for Health Research (KPCHR) at Kaiser Permanente Northwest (approval ID: MOD20050381-003), where the study was conducted. Interviews were conducted via KPCHR Teams, and digital recordings and transcripts were securely stored within the primary organization’s firewall-protected file service.

### Data Collection

Semistructured interviews were conducted remotely via Microsoft Teams. The interview guide (Multimedia Appendix 1) was developed by our medical anthropologist expert (NV) in coordination with the principal investigator (FL). Additionally, a qualitative investigator from the larger BritePath team reviewed the guide for content. The interviewer (FL) used a semistructured interview guide (Multimedia Appendix 1) to ensure consistency across interviews. The framework that guided the design of our qualitative interview guide focused on usability and concept testing. We examined whether providers from different backgrounds and training would consider using BritePath. The included questions aligned with several frameworks for adopting new technology, such as the Diffusion of Innovation theory [26,27]. The interviews assessed providers’ opinions on using apps with patients and their impressions of the benefits and barriers of mental health apps in general. Participants were then shown a series of visuals (screenshots) depicting what a user would see while interacting with the BritePath app. These prompts ensured that participants had a clear and consistent understanding of the app’s features and user interface. During this portion of the interview, participants were asked about their initial impressions of the BritePath app, as well as the barriers and potential benefits of using it in their practice. They were also given the opportunity to suggest features they would like to see in future app developments. Each interview lasted 30-40 minutes and was audio recorded and transcribed by an independent transcriptionist. Transcripts were entered into Atlas.ti [28], a qualitative analysis software program used for coding and managing data. Atlas.ti was then used to generate reports for the analysis of interview transcripts.

### Analysis

Thematic analysis, a method for analyzing patterns of meaning in a data set, was used to interpret the data by identifying themes and their interconnections [29-32]. Following established thematic analysis procedures [30], members of the research team (JC, LF, and NV) first familiarized themselves with the data and, together with FL, developed an initial code list consisting of a priori codes reflecting the interview questions. JC and LF coded the first interview using this initial code list and identified additional codes while reviewing the transcripts. The full team met to review coding consistency between reviewers and to evaluate the need for new, emergent codes. Modifications to the code list were made based on discussion and consensus. This process was repeated, with 2 team members coding 2 additional transcripts each. The full team met to discuss differences between coders for these additional transcripts and found minimal discrepancies in the codes applied. Upon confirming sufficient consistency in code usage and identifying no additional emerging codes, a master code list was finalized and used to code the remaining transcripts. After coding all transcripts, the full study team individually reviewed reports of coded interview segments to identify themes. The final themes were then derived through discussion and consensus. This formal, team-based analysis procedure was used to enhance the credibility and trustworthiness of the findings.

## Results

### Providers’ Perspectives on Mental Health Apps and the BritePath App for Youth at Risk of Suicidal Behavior

We conducted semistructured interviews with a convenience sample of 17 providers from 7 states in the United States (Table 1). Our goal was to understand providers’ opinions on the use of apps for youth with mental health concerns in general and the use of the BritePath app for youth at risk of suicidal behavior. First, we asked providers about their experiences using mental health apps with patients. Next, we gathered their opinions on a specific app, BritePath, designed to improve patient engagement and communication with providers for youth at risk of suicidal behavior. The interview guide is available in Multimedia Appendix 1.

**Table 1:** Provider demographics (N=17).

Characteristic		Values, n (%)
**Age (years) group**		
	25-34	4 (23)
	35-44	6 (35)
	45 and over	7 (41)
**Sex at birth**		
	Female	15 (88)
	Male	2 (22)
**Provider type**		
	Physician	2 (22)
	Therapist/social worker	8 (47)
	Psychologist	7 (41)
**Type of setting**		
	Large health system	10 (22)
	Smaller group practice	4 (47)
	Individual practice	3 (41)

### Providers’ Opinions About the Use of Apps for Mental Health Concerns in General

#### Overview

All providers reported familiarity with mental health apps, and most stated that they had referred at least one patient to a mental health–focused app—for example, to help patients learn a skill such as relaxation. However, none of the providers reported regularly using any mental health app during treatment visits or in an interactive manner with patients. Each theme is discussed in more detail below.

#### Theme 1: Mental Health Apps Have the Potential to Be Valuable as an Adjunct to Therapy Visits

Providers reported that they felt mental health apps could add value to the therapeutic relationship as an adjunct or complement to face-to-face or video-based psychotherapy sessions. However, most did not believe that mental health apps could serve as a substitute for psychotherapy visits. Several providers noted that these apps can offer access to exercises for learning new skills or encourage the use of tools discussed in therapy, making them a useful complement to treatment sessions.

I think they can be really great adjuncts if you have the time to utilize those as a clinician. I am always interested for my patients of course for them doing things in the many hours outside of therapy, so I see you from 1:00 to 2:00 and then there are all these other hours that in theory you’re doing the things that we were practicing in that one hour. If there are apps that can promote, engage, and promote the use of those strategies, whether it’s thought records or sleep diaries or mood meters, things like that. And then they can bring that back in and we can look at it together and that facilitates the conversation, I think that would be great.

P5

And this way as a clinician I am teaching a skill, but I want it to be reinforced by this tool that mainly my client really loves but eventually is a way to train the brain to think a new and different way.

P4

Providers also noted that this approach to learning skills may be particularly useful for teenagers, who frequently engage with their phones.

I think they’re a great compliment to therapy. I think they’re really useful as a tool. I am not a huge fan of the app-based therapies you know. Like text therapy and stuff like that, I think it’s not a good fit for everybody of course. But I think the apps themselves as far as tools and teaching and reinforcing skills I think are really helpful. And I would imagine for teenagers, I am assuming most of them are on their phones quite a bit. So that might be kind of a really easy transition or way to incorporate into something they’re already doing. I think it would be cool especially for teens if there was a way to have some sort of feedback or back and forth with their therapist so they could actually share what they’re doing, that would be neat. I think there’s a lot of potential there for younger people.

P14

Although providers generally expressed support for mental health apps, most also raised several concerns. They described issues from both the provider and patient perspectives.

#### Theme 2: Cost/Access to Tool

The cost of purchasing an app was noted as a potential barrier. Providers suggested that they would only consider recommending apps with relatively low fees or a 1-time payment rather than ongoing costs.

Obviously free is best. Especially for teens, even young adults that are trying to make ends meet. If not then maybe something that’s a one-time fee. If it was a subscription that would be harder for that population. I think that’s something that older adults could possibly swing, but unless the parents are paying for it, it doesn’t seem like that would be a good match.

P13

On the patient side yeah I think it would depend on how expensive the app would be. If they’ve got to pay $50/month that certainly could be prohibitive for folks.

P5

In addition, providers expressed concern that some youth, particularly those from low-income backgrounds, might lose phone service or have limited minutes, which could hinder access to the app and reduce engagement with its tools. They also noted that youth might have only intermittent access to mental health apps for various reasons, such as an inability to consistently pay for phone service or depleting their data or minutes. This loss of access could be frustrating for youth trying to use mental health apps and could interfere with their engagement with the app’s content.

Most of our patients do have smart phones so that’s usually not a barrier. But sometimes getting their cell phones shut off has happened before. So whether or not they’ll have continuous access I think has been a barrier in the past.

P8

#### Theme 3: Engagement

Many providers expressed concern that a common barrier to using mental health apps is a lack of patient engagement. Several participants shared experiences of patients downloading apps but never using them beyond the initial session. Others noted that apps often lose their appeal quickly, making sustained engagement a challenge.

Yeah, and I think the literature is pretty clear that if you send patients off to use some sort of behavioral health app, they'll use it once or twice. They'll check it out and then use drops of precipitously.

P1

I think patients often download and forget about them which is probably the biggest one is the engagement with some of these apps is low. After a short amount of time they might forget about it and then they don’t really come back to it. So in the context of therapy that was less of an issue because I would be there and be encouraging that use. So that’s one of the barriers if people were just using it out in the real world.

P9

Providers described challenges in getting youth to engage with an app at all. Several noted concerns that apps must be visually appealing and up-to-date to attract teens, who are often highly tech-savvy. Based on their experience, if an app was not engaging enough, usage would decline, similar to what they had observed with other patient-focused apps.

I think we all know that teens are very sophisticated with apps and tech so I think having it be interesting and up to date is I think if they’re going to use it, it’s got to be flashy in some way.

P3

#### Theme 4: Patient Privacy

Most providers expressed concern about patient privacy. Some noted that their patients had raised concerns about the privacy of data collected by apps, which could make them hesitant to use mental health apps. Providers also emphasized that health systems would need to ensure patient privacy before integrating an app into treatment.

I know so many patients just in general are very concerned about their privacy. So I think that would be at the top of my list is just knowing that I could have confidence in saying to this person this app has been vetted in these different ways. This is the security that it offers. Maybe it's even password protected when you sign in so that way if your mom picks up the phone and she can't get into that particular app. I feel like that's a deeply personal thing. And I would want to make sure that I am protecting my patient's confidentiality and I can speak to how this app does that.

P2

I think always a concern is privacy and making sure the data is secure and HIPAA compliant. Especially working in health systems we’re always concerned about technology and the privacy of patient information.

P9

Some providers also discussed concerns about patient privacy at home. Before recommending an app, they wanted to be confident in their understanding of how patient data were being protected.

On the other hand, if their parents are checking their phone and they don’t feel like they have privacy they might not be able to use it in the same way that they might have otherwise had they had more privacy.

P8

### Providers’ Opinions About the Use of the BritePath App

#### Overview

All of the themes providers discussed regarding mental health apps in general were also reported in relation to the BritePath app. For instance, providers expressed privacy concerns both broadly and specifically in the context of BritePath. After hearing a description of BritePath, all but one provider expressed interest in trying this type of app for patients with depression, suicidality, or both. In addition to the general themes identified for mental health apps, several additional themes emerged specific to BritePath.

#### Theme 1: May Make Visits More Efficient

Providers described several ways in which an app such as BritePath could make therapy visits more time-efficient. Some noted that BritePath could help patients gain insight into the relationship between their mood and the activities they engage in between visits.

I think such a common thing with younger teens is being able to reflect back on how their week went or how their time went. And often times having something that is kind of tracking in the moment gives so much more actual information of when there were any spikes in anxiety or in distress in some way to help be like oh I saw this happened on this day, what happened then. To help anchor them into being able to talk about some things. So I can see that being a real benefit.

P15

I think it’s so important for people to be able to see visually what the distribution is of the moods throughout the week. And also to remember that they’re not always in that low point. So having some check ins throughout and maybe not only on their really bad days, right, but check ins every day would be helpful probably so they could be able to track down only the downs but maybe the ups or the little bit better.

P14

BritePath was also seen as a tool for providers to gather information on patient moods and their use of techniques between in-person sessions. Providers noted that access to this information via the app could make sessions more focused and productive by reducing the need to collect it during visits and allowing for better session preparation. Additionally, real-time patient-reported data on the app were perceived as more complete and valid than recalled information shared during a visit.

If you had the time to review what someone did or didn’t do since you last saw them before they even walked in the door...I’ve saved at least 10 minutes of the precious 45 that we have together. I can launch straight into hey your mood meter was great over the last week. Or I see you check in on your safety plan on Tuesday, tell me a little bit about that? I don’t have to say so how did the last week go? Oh Tuesday was bad. Tell me more about Tuesday and then wait for them to bring up the fact that they had to use their safety plan on Tuesday. We can just get straight to it. And it helps...not only the time saver, but hopefully not having to deal with retrospective recall.

P5

I think too if we have a session with a patient and they’re just in that moment trying to recall their week is not as impactful as if you can actually go back and see what they’re feeling in any particular time and probably would really help with recall bias.

P8

Providers also discussed how the BritePath app could facilitate communication with youth and enhance their engagement in the treatment process.

I think...leveraging digital health tool...I think another place this can be helpful is if kids engage in it, it gives you more to talk about in the session. You know doing sessions with teenagers a lot of times it’s pulling teeth to get them to say anything. At least this would provide them with one, another way to keep them engaged and two, more fodder for talking to someone that may not be interested in filling 45 minutes’ worth of talking.

P5

#### Theme 2: Provides Access to Safety Plans

Most providers noted that a key benefit of BritePath was giving patients convenient and consistent access to their safety plans through the app. They believed that because most youth carry their phones, they would be more likely to have their safety plan readily available in a crisis—unlike a paper version, which could be easily misplaced.

I think the nice thing about it, is it’s all in one place. It’s easily accessible on our phone. If they’re in distress at any given moment they can pull it out and it’s all right there. That makes it much more likely that they’re going to use their skills in the moment, versus us giving them a piece of paper or emailing them or whatever else technology that would be used in the past. So I think that’s a huge advantage.

P13

So patients can have a safety plan in the EHR but if they can't access it. We print it out and who knows where they left it. This gives them something, most people are pretty good about not leaving without their phone. So, it's a way that's there, it's accessible.

P1

However, providers also had questions and concerns about the functionality of storing the safety plan on a youth’s phone. Some wondered whether the safety plan could be easily integrated into the patient’s electronic health record. Another concern was whether providers would be notified when a patient accessed or used their safety plan.

Would the provider be alerted if there was a change? For example, if someone were to use the program or use part of their safety plan would that then ping me as a provider? Or would I just need to go and check on the patient status every so often to know those sorts of things? Another thing I might be curious about is how it might communicate with my EHR, if there was a way to import the information or download, even the safety plan for example so that way I could then upload it into the patient's chart. I think that would be important for me too.

P2

#### Theme 3: Offers an Additional Way to Communicate With Patients

Providers noted that BritePath could serve as an alternative communication tool for patients. Some recounted experiences with teens who struggled to express themselves and suggested that BritePath could offer these youth another way to connect with their providers. One provider also noted that this feature could enhance engagement in treatment for some patients.

Obviously, someone who is busy, if they’re actually busy or just having issues with talking with a clinician face to face, this is a way to avoid that stress of direct interpersonal interaction. So that’s a positive. And adolescents have all sorts of reasons why they sometimes don’t want to engage with care. This is a way for them to get some care even if they’re not feeling like interacting with their clinician that week. That I see as a benefit. Asynchronous so it can be on their time rather than someone else’s time.

P12

If you think about teenagers, they are much more comfortable with technology and sometimes that’s a great place to start for therapy or treatment with them because they might not feel comfortable talking to a professional.

P9

Several providers valued BritePath’s 2-way communication feature, noting that it could facilitate shared decision-making and provide real-time updates to the treatment plan.

I think there's a real value in that shared decision-making component that can go into an app like the one you're describing. And it also becomes an important way of sort of communicating.

P1

I think that two-way communication is what I think could be the most useful if it’s going between the patient and the therapist and you’re able to interact back and forth. So in terms of modifying treatment plan or knowing what is resonating with the person.

P3

#### Theme 4: Flexibility and Personalization of the BritePath App

A number of providers expressed enthusiasm for BritePath’s flexibility and personalization features. Several noted that allowing youth to customize the app with photos, videos, motivational reminders, or prompts for coping strategies could help sustain engagement, increase usage frequency, and accommodate diverse patient needs.

I think that’s definitely a bonus. Sometimes the out of the box app works just fine. But the more you can tailor it or personalize it to someone’s specific needs, the more likely they are to use it, so I think that that could definitely be useful!

P9

I love that there can be general tools but also some really individualized personal things in there in too. I just love the idea of having names and photos of people, loved ones and pets and things like that.

P14

One provider highlighted BritePath’s potential to empower youth by allowing them to personalize their treatment and safety plan.

The place a teen usually goes to for soothing support regulation is the phone. But it often feels like the phone is just riddled with pitfalls of things that could make things worse. Maybe don’t go on Instagram and compare your life to other people. So the fact that there’s something right next to it potentially on their phone for them to dive into instead of something that could be potentially more disregulating or harmful I think is fabulous. The more opportunity I think there is to customize what’s happening in that app the more I’d be inclined to really empower my client to tap into a wise place, tap into wiser parts of themselves, have those places and parts load what’s in the app. So then they have access to it in those harder moments. I could see that being a particularly empowering thing.

P11

The collaborative functionality of the app was also seen as a benefit, as it enables providers to modify the app’s content in collaboration with patients over time as their needs change.

I think that two-way communication is what I think could be the most useful if it’s going between the patient and the therapist and you’re able to interact back and forth. So in terms of modifying treatment plan or knowing what is resonating with the person.

P3

#### Theme 5: Concerns About Integration of BritePath Into Clinical Workflow

Providers expressed additional concerns specific to BritePath, beyond those related to mental health apps in general. One concern was its impact on clinical workflow. For instance, providers questioned how well the app would integrate with existing electronic medical record systems. They also expressed concerns about its ease of use for busy clinicians.

Another thing I might be curious about is how it might communicate with my EHR, if there was a way to import the information or download, even the safety plan for example so that way I could then upload it into the patient's chart. I think that would be important for me too.

P2

Do I have to have a certain log in? Do I see a list of patients that I have that are using it? How cumbersome is it to get into that? And what's the patient's understanding and expectations for me, either monitoring that data or accessing that data or is there even some sort of portal to do that? Or is it more that the patient brings in their phone with their smart phone app and sort of shows me what they've been up to?

P1

In some cases, these concerns amplified existing worries about mental health apps in general, such as what might happen if a patient experiences a crisis. Additionally, several providers raised concerns about managing patient expectations regarding 24/7 access to their provider and ensuring that patients understood how to handle crises when providers were unavailable.

The only thing that has come up before... as far as cell phone and texting technology is somebody trying to text you in crisis while you’re off work. You’re not checking, so if there’s something that presents in the app that is alarming or suggests that this client is in danger of harming themselves and you haven’t checked the app in whatever amount of time, that would be I guess the concern for me too.

P13

I would want to make sure that whatever the app is that it stays within the boundaries of how I am available to the client or not. And I’d want it to be clear, it’s tricky because if I can get a report about where they’re at conceivably I could get a report when they’re having a moment of increased distress and does that require me to then reach out to them too? So, I’d want it set up to be like hey either this app only releases this data to me X amount of times, so if you put in that you're having a hard time I am not going to see it.

P11

## Discussion

### Principal Findings

The focus of this qualitative study was to gain a deeper understanding of provider opinions on the use of mental health apps in general and the BritePath app specifically for treating teen and young adult patients with depression, suicidality, or both. Providers in this study had no prior exposure to the BritePath app. Most were aware of mental health apps in general and expressed interest in trying the BritePath app with patients with depression, suicidality, or both. Nearly all providers viewed mental health apps as a complement to other mental health treatments rather than a replacement for psychotherapy visits.

### Mental Health Apps in General

Most providers were supportive of mental health apps, recognizing their potential to provide access to information and skills that could improve youth mental health. Key benefits included their availability at any time—particularly when providers were not accessible, such as late at night—and their ability to reinforce skills such as stress reduction between sessions. However, providers differed in their level of comfort with these tools. Some frequently recommended resources such as online cognitive behavioral therapy programs or anxiety management apps, while others were less enthusiastic. Most providers noted that mental health apps might be particularly useful for younger individuals, as they frequently use phones and other digital devices. These findings align with existing research on provider and patient perspectives, which indicate broad support for digital mental health tools [13,17,33-35]. However, most providers emphasized that apps alone were not sufficient for youth with significant mental health concerns. Many explicitly stated that these tools should complement, rather than replace, psychotherapy.

All providers expressed concerns about patient privacy. They discussed potential risks related to data collection by apps, including unauthorized access by individuals other than the youth (eg, parents) and uncertainties about how app providers might use collected data. While privacy concerns are common with app usage in general [13], providers emphasized that data collected by mental health apps are particularly sensitive and require high standards of privacy and protection. Several providers noted that app usage has become so commonplace that privacy policies are often overlooked, which may be especially problematic for youth. One provider specifically highlighted the difficulty in determining how commercial apps safeguard data privacy, which they found concerning.

Many providers also raised concerns about maintaining youth engagement with apps. Difficulty in sustaining engagement with digital tools has been noted in prior studies [36,37]. Several providers pointed out that they currently have little to no insight into how engaged their patients are with mental health apps. This uncertainty tempered some providers’ optimism about the potential of these tools as a strong aid in treatment.

Providers also identified several logistical barriers to the effective use of mental health apps. The cost was a common concern, particularly the potential inaccessibility of apps that require ongoing payments (eg, monthly fees). Additionally, several providers noted that youth often experience inconsistent access to phones due to service suspensions, late payments, or lack of internet access. While these concerns have been raised in studies on app use in general [13], they may be especially critical for youth with mental health concerns, as intermittent access could disrupt their ability to use these tools as a coping mechanism.

### BritePath App

Each of the potential benefits and concerns providers expressed about mental health apps in general were also raised in relation to BritePath, including data privacy, patient engagement, and cost-related concerns. In some cases, providers expressed stronger opinions—both positive and negative—specifically regarding BritePath. For example, concerns about patient privacy were heightened due to the app’s inclusion of sensitive information on symptoms and suicidality. By contrast, providers strongly supported BritePath’s potential to enhance psychotherapy visits by facilitating more in-depth collaboration between providers and patients on skill development.

Providers raised several additional themes regarding BritePath. Many highlighted the value of the app’s personalization features, noting that these could enhance patient engagement. Most providers expressed enthusiasm for BritePath’s ability to facilitate communication between sessions and help providers prepare more effectively for upcoming appointments.

Providers also raised concerns about ensuring that safety issues were thoroughly addressed if BritePath were to be used in practice. While they appreciated the idea of having a safety plan integrated into the app, they emphasized the need for built-in safeguards, such as pop-up messages directing patients to emergency services when necessary. Additionally, providers wanted to ensure that patients understood that communication through the app would not guarantee a 24/7 response from providers.

### Practical Implications for Research and Clinical Practice

Providers suggested several ideas for future research on apps addressing suicidality in youth. Integrating ambient phone data, such as activity tracking, could enable more timely and accurate recording of safety plan activities, reducing reliance on self-reporting. Additionally, visually linking mood and activity data, along with incorporating pop-up mood assessments, could enhance patient awareness of the relationship between mood and behavior. Increasing personalization in mood tracking and allowing patients to annotate their mood data may further improve engagement with the app.

Although providers were enthusiastic about the potential benefits of a customizable, readily accessible safety plan on a patient’s phone, many also raised concerns about ensuring the security of this information. From a clinical perspective, safeguarding safety plans on the device would be essential for successful implementation within health systems. Additionally, embedding a clear and transparent patient consent process into the app—one that explains who will have access to their data and why—would be crucial in helping patients understand the risks associated with using a tool such as BritePath. Furthermore, ensuring that BritePath is compatible with different phone operating systems and can integrate with various electronic health record systems would be key to facilitating widespread adoption in clinical practice.

### Strengths and Limitations

This study provides new insights into providers’ perspectives on the use of mental health apps for youth at risk of suicidal behavior. We acknowledge the potential for sampling bias. To address this, we aimed to recruit a diverse range of providers from different health systems affiliated with the NIMH Mental Health Research Network [23]. While this sample may not be fully representative of providers across all health systems, participants were drawn from health systems in 7 US states. Although the sample is not fully representative of all providers in the United States, it includes a variety of geographic areas, organizational arrangements, and provider types, ensuring a diverse range of perspectives. In designing our qualitative interview guide, we focused on usability and concept testing rather than adhering to a specific technology adoption theory, such as the Technology Acceptance Model [26] or the Diffusion of Innovation Model [27]. However, our interview questions covered many of the key domains included in these models. We invited providers who delivered mental health services within these health systems to participate, without requiring a specific level of experience with apps or other digital tools in their practice, as we aimed to capture a broad range of experience levels. It is possible that providers with a greater interest in app use were more likely to participate.

One goal of this study was to explore providers’ opinions about the BritePath app, which was developed for use with youth at risk for suicide. Specifically, we sought feedback from providers with no prior clinical experience or knowledge of BritePath to assess its potential for future implementation in health systems. Our focus was on understanding the broader applicability of mental health apps, with a particular emphasis on BritePath. While we collected limited demographic data on providers, it is possible that unmeasured factors influenced their perspectives on the use of mental health apps. For example, although respondents represented a range of age groups, most were younger providers, who may have been more comfortable with apps in general than older providers. Despite these limitations, this study offers new insights into providers’ perspectives on mental health apps, including the BritePath app, and highlights key concerns regarding their use with young patients at risk for suicide.

### Conclusions

The use of mental health apps is expanding, yet more research is needed to determine how they can be most effectively integrated into mental health treatment. Most providers expressed interest in using the BritePath app for patients with depression, suicidality, or both; however, concerns about privacy and safety remain.

## Appendix

Multimedia Appendix 1 Qualitative interview guide.

## Abbreviations

ETUDES Center: Center for Enhancing Treatment and Utilization for Depression and Emergent Suicidality
KPCHR: Kaiser Permanente Center for Health Research
NIMH: National Institute of Mental Health
