# Supplementary material for: Provider Perspectives on the Use of Mental Health Apps, and the BritePath App in Particular, With Adolescents at Risk for Suicidal Behavior: Qualitative Study
Source: JMIR Hum Factors. 2025 Feb 26;12:e64867. doi: 10.2196/64867 (PMC11904364; doi:10.2196/64867)
Supplement: Multimedia Appendix 1 [file humanfactors_v12i1e64867_app1.docx]

Etudes study provider interview guide January 20, 2020

Introduction: Hi, my name is [Interviewer name], and I’m from the Kaiser Permanente Center for Health Research. Thank you for taking the time to help us. Is this still a good time to talk?

Our conversation today will help us learn your thoughts about use of new technologies, such as apps, to support your patients, particularly teens with depression or who are at-risk for suicide. We will ask you some general questions, and then ask you about one specific app that currently is being tested in a clinical trial. The University of Pittsburgh and the Kaiser Permanente Center for Health Research are participating in this trial. The purpose of this app is to connect depressed and suicidal adolescents with mental health treatment. Currently, in primary care the rate of connecting these teens to mental health treatment is low, and we are testing interventions that aim to improve referral and treatment engagement rates.

During the interview we want to learn from you about your experience with using apps for treatment and more importantly the potential benefits and disadvantages you see in using such apps in primary care for teens. We expect the interview to take 20-30 minutes.

I will be recording the interview to make sure I don’t miss anything. Everything you tell me today will be kept confidential. The recording will be transcribed in a way that removes all your identifying information. In future publications we may use any quotes that explain a point particularly well, but nothing we use will identify you personally in any way. You do not have to answer any questions you don’t want to, and you can stop at any time.

Do you have any questions before we get started?

Do I have your permission to record our interview? [IF PARTICIPANT DOES NOT GIVE PERMISSION, STOP INTERVIEW. If participant gives permission, start recording. Repeat after recording has started that you have participants’ permission to record interview].

-------------------------------------------------------------------------------------------------------------------------------

OK the recorder has started, so just for the record, do I have your permission to record this interview?

**Let’s start by talking about any apps you may have used in the past to provide care/monitor/communicate with patients?**

**Prompts:**

What specific apps did you use with patients?

What was that experience like?

Why did they use it, specific barriers they encountered?

Barriers related to patient or provider time?

**In your opinion, what role should apps play in providing care to teenage patients and communicating with them?**

**Prompts:** e.g. serve as communication channel, complement treatment, replace treatment

**(*Describe BRITEPath APP*** *(Read by interviewer))***: “BRITEPath** is an intervention aimed at guiding mental health clinicians through the process of onboarding a safety plan onto the BRITE app and follow patient symptoms and use of their safety plan on the app between sessions.  The intervention is aimed at allowing most professionals “off the street” to develop a safety plan with little training.”  To give you a feeling for BRITEPath, I’m going to show you a couple of screen shots from the app. The first one shows a mood rating tool that patients are asked to do every day, or as frequently as they are willing.

*(Show Screen shot to participant)*


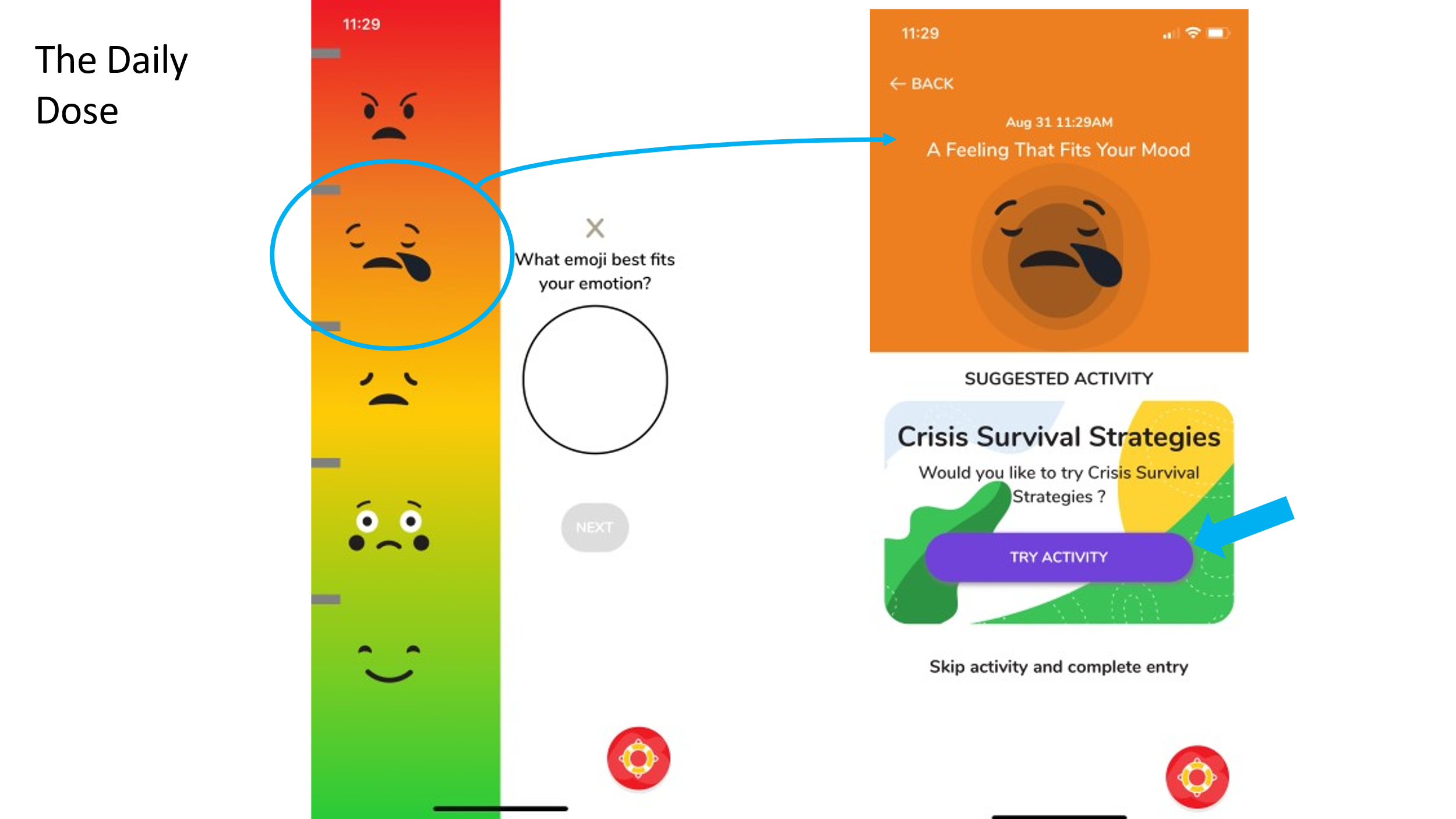


The next slide shows an example of how a provider would build a safety plan with the patient, including resources the provider might recommend, and skills that the provider might want to have the patient learn. *(Show Screen shot to participant)*


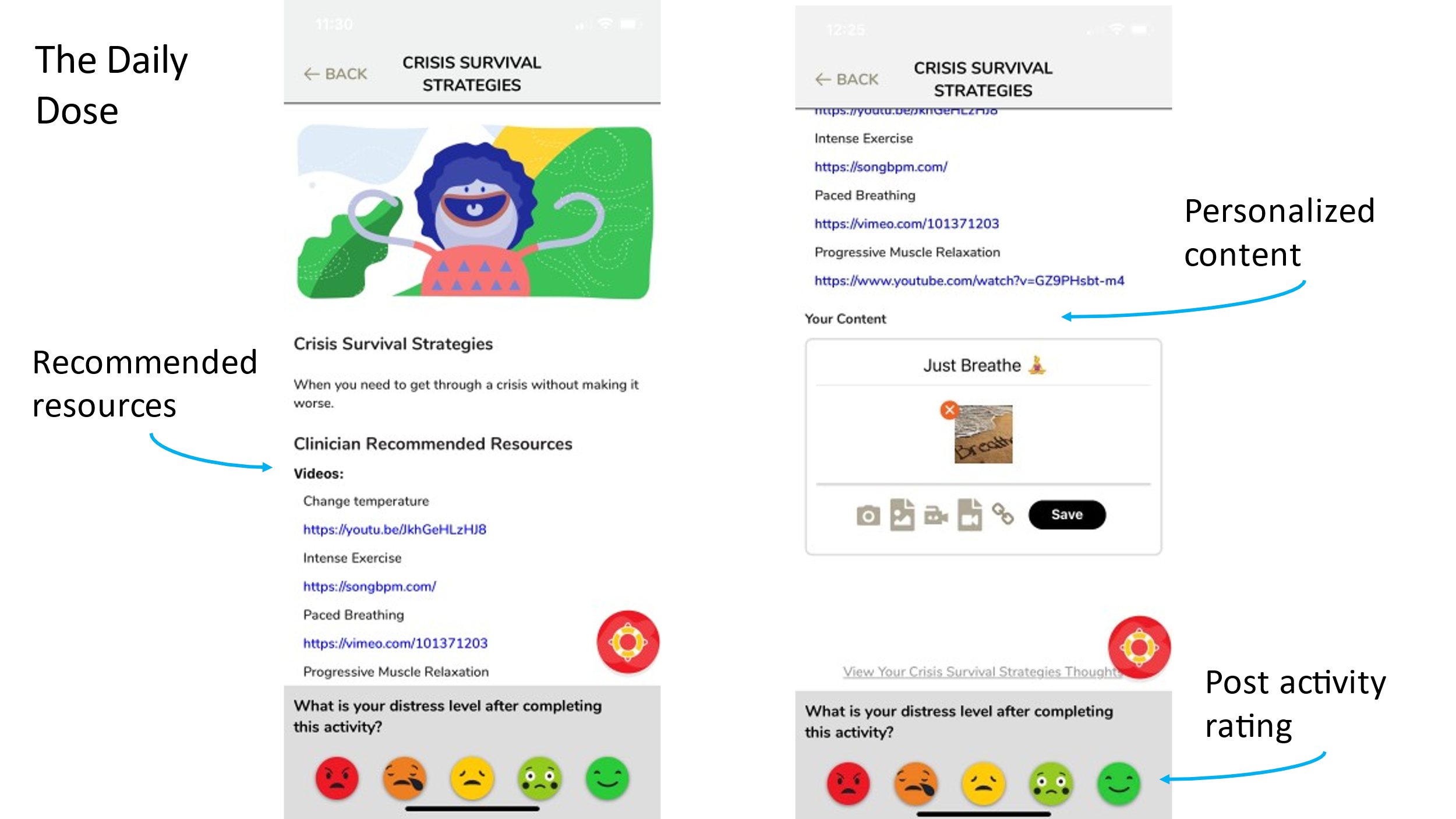


The final part of the app I’d like to show you is the online dashboard. The clinician uses an online dashboard to follow symptoms, connect coping skills with mood ratings by the patient and increase ability of clinicians to use measurement-based care approaches through sending regular self-reports directly to patient’s app.


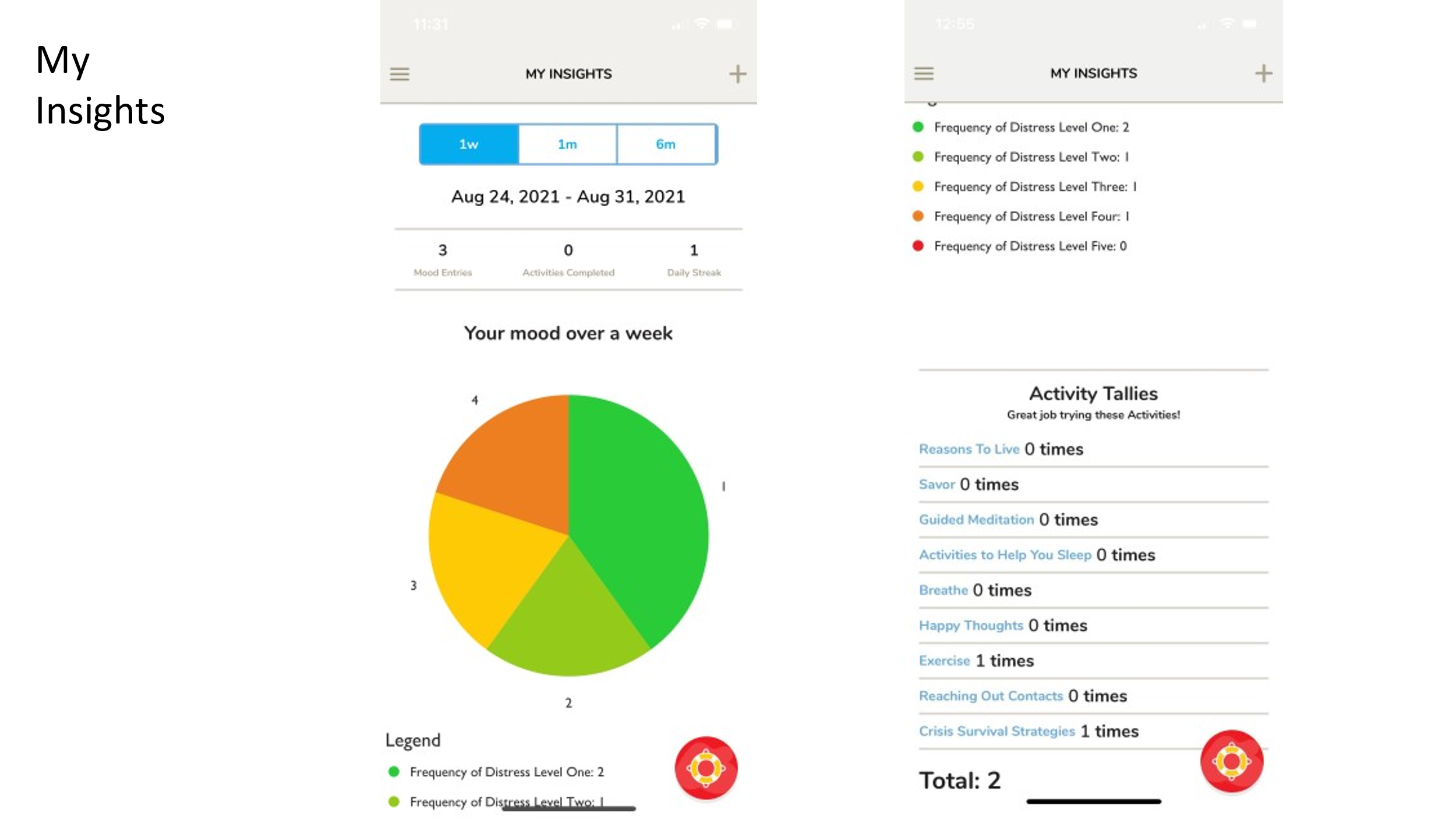


Thinking about adopting THIS TYPE OF APP that I described just now, would you be interested in using this app in your practice? Why or why not?

How important is the availability of scientific evidence about the effectiveness of this app in considering using it?

What other aspects are important for you when considering using the app? Do you think you might use it?

**Prompts:** Training, tech support, patient interest, fits with the type of therapy you do?

What additional information might you need to be able to make an informed decision?

Prompt: How important is it to tailor an app to the specific needs of a patient?

We’re interested in potential barriers to adopting a tool like this for treating teens with depression/suicidality. Are there particular barriers that you anticipate? What are those barriers?

*(Then prompt for any of the following: [if they mention past experiences while going through this list, follow-up to ask about specific information])*

**Prompts:**

Learning/using these strategies might be time-consuming and demanding.

Concerns about provider needing to teach the skills in the safety plan (e.g., relaxation) – time, scope of training

Concerns about using these new tools to influence the clinical decision-making process.

Concerns about any legal liability related to using these tools.

Administrative and institutional requirements or limitations.

The current reimbursement structure does not incentivize using these methods.

Concerns about fit with style of treatment/therapy the provider uses

Concerns about fit with the patients’ goals, or what they already currently do (e.g., communication on social media)

Other financial/cost considerations?

Are there any advantages or facilitators that you think could make an app like this a useful tool?

**Prompts:**

Identify patients who may need additional mental health services in a more timely manner

Decrease patients’ fear and stigma associated with mental health treatment.

Improve access to mental health care.

Allow patients to be more committed to and engaged with their mental health treatment.

Improve patients’ adaptive behavior and functioning.

Promote the supportiveness of the caregiver/family with this teen.

Please tell me about any other factors not already discussed that should be considered in adopting tools like this for clinical use.

Thanks for taking the time to talk with us today.
